# Physical Exercise Methods and Their Effects on Glycemic Control and Body Composition in Adults with Type 2 Diabetes Mellitus (T2DM): A Systematic Review

**DOI:** 10.3390/ejihpe13110176

**Published:** 2023-11-05

**Authors:** Bastián Parada Flores, Pablo Luna-Villouta, Cristian Martínez Salazar, Jorge Flández Valderrama, Luis Valenzuela Contreras, Carol Flores-Rivera, Rodrigo Vargas-Vitoria

**Affiliations:** 1 Facultad de Educación, Magíster en Ciencias de la Actividad Física, Universidad Católica del Maule, Talca 3460000, Chile; paradafloresbastian@gmail.com; 2 Facultad de Educación, Departamento de Educación Física, Universidad de Concepción, Concepción 4030000, Chile; pabloluna@udec.cl; 3 Departamento de Educación Física, Deportes y Recreación, Universidad de La Frontera, Temuco 4780000, Chile; cristian.martinez.s@ufrontera.cl; 4 Facultad Filosofía y Humanidades, Instituto de Ciencias de la Educación, Escuela de Educación Física, Universidad Austral de Chile, Valdivia 5090000, Chile; jflandez@uach.cl; 5 Facultad de Educación, Pedagogía en Educación Física, Universidad Católica Silva Henríquez, Santiago 8330226, Chile; lvalenzuela@ucsh.cl; 6 Facultad de Educación y Ciencias Sociales, Universidad Andres Bello, Concepción 4030000, Chile; carol.flores@unab.cl; 7 Facultad de Educación, Pedagogía en Educación Física, Universidad Católica del Maule, Talca 3460000, Chile

**Keywords:** T2DM, glycemic control, body composition, physical exercise

## Abstract

The prevalence of T2DM represents a challenge for health agencies due to its high risk of morbidity and mortality. Physical Activity (PA) is one of the fundamental pillars for the treatment of T2DM, so Physical Exercise (PE) programs have been applied to research their effectiveness. The objective of the study was to analyze the effects of PE methods on glycemic control and body composition of adults with T2DM. A systematic review without meta-analysis was performed, using the PubMed database. Quasi-experimental and pure experimental clinical trials were included, which were available free of charge and were published during 2010–2020. In the results, 589 articles were found and 25 passed the inclusion criteria. These were classified and analyzed according to the methods identified (AE, IE, RE, COM, and others), duration and variable(s) studied. It is concluded that PE is effective for glycemic control and body composition in adults with T2DM using different methods (AE, IE, RE, COM, and others), both in the short and long term. Adequate organization of PE components such as frequency, duration, volume, and intensity, is essential.

## 1. Introduction

The prevalence of Diabetes Mellitus (DM) is high worldwide and represents a major challenge for health agencies, due to its high risk of morbidity and mortality [1,2]. According to figures from the “*International Diabetes Federation (IDF) Diabetes Atlas 9th edition 2019*” [2], it is estimated that DM affects 9.3% of the world adult population, that is, approximately 463 million people, while projections for 2030 and 2045 are 578 and 700 million, respectively. In Chile, it is estimated that 1,262,200 people between 20 and 79 years of age have DM, which determines a prevalence of 8.6% in the adult population, of which 90–95% corresponds to Type 2 Diabetes Mellitus (T2DM), and it is projected to increase to 9.8% by 2030 and 10.4% by 2045.

The increase of this disease causes an important individual, social, and economic impact. According to the IDF [2], it is estimated that 11.3% of deaths worldwide are due to T2DM, and approximately 50% of these cases are people under 60 years of age. In economic terms, about 10% of the annual world health budget is spent on treatments for T2DM and its associated complications, i.e., about 760 billion dollars, while in Chile this expenditure would be about 1770 million dollars. To this, indirect costs are added, generated by premature death and disability, which in turn cause a decrease in labor productivity, and intangible costs such as the worries and discomfort of living with this disease.

T2DM comprises between 90–95% of the total cases of diabetes and its main risk factors are excess body weight and physical inactivity [1], about 74.2% of Chileans are overweight [3] and the prevalence of a sedentary lifestyle is 87.3% [4].

The World Health Organization (WHO) emphasizes that physical activity (PA) is one of the fundamental pillars for the treatment of T2DM, along with diet and medication, and, therefore, a minimum of 150 min of moderate-intensity aerobic PA or 75 min of vigorous aerobic PA per week is prescribed for adults [1]. Likewise, the American Diabetes Association (ADA) [5] suggests the incorporation of recreational PA and flexibility and balance exercises into the daily routine. However, prevalence figures demonstrate the need to vary methods.

Several studies [6,7,8] have reported significant benefits of systematic PA, developed as physical exercise (PE) programs, in stabilizing plasma glucose and improving adiposity and muscle mass parameters of individuals with T2DM, which is related to the physiological adaptations induced by PE, which favor insulin sensitivity and metabolization and optimization of metabolic reserves [9]. In this context, the relevance of this research focuses on investigating the literature on the effects of different PE methods on glycemic control and body composition in adults with T2DM, and aims to define how PE components affect the treatment of this disease, based on a systematic review.

## 2. Materials and Methods

The study corresponds to a systematic review without meta-analysis, with a research design based on the protocol of the PRISMA-P 2015 statement. All articles included are original and correspond to primary studies, theses, and dissertations. Systematic reviews with or without meta-analysis were excluded.

### 2.1. Search Strategies

The PubMed electronic database was searched, using advanced filters to find types of studies, periods of publication, and specific languages. The search comprised key concepts filtered with MeSH terms and linked to Boolean operators, completed as follows: (((((((type 2 diabetes[MeSH Terms]) OR (diabetic patients[MeSH Terms])) AND (physical exercise[MeSH Terms])) OR (training[MeSH Terms])) AND (glycemic control[MeSH Terms])) OR (glucose[MeSH Terms])) OR (HbA1c[MeSH Terms])) AND (body composition[MeSH Terms]), and its Spanish and Portuguese language counterparts.

### 2.2. Selection of Studies

**Figure 1:**
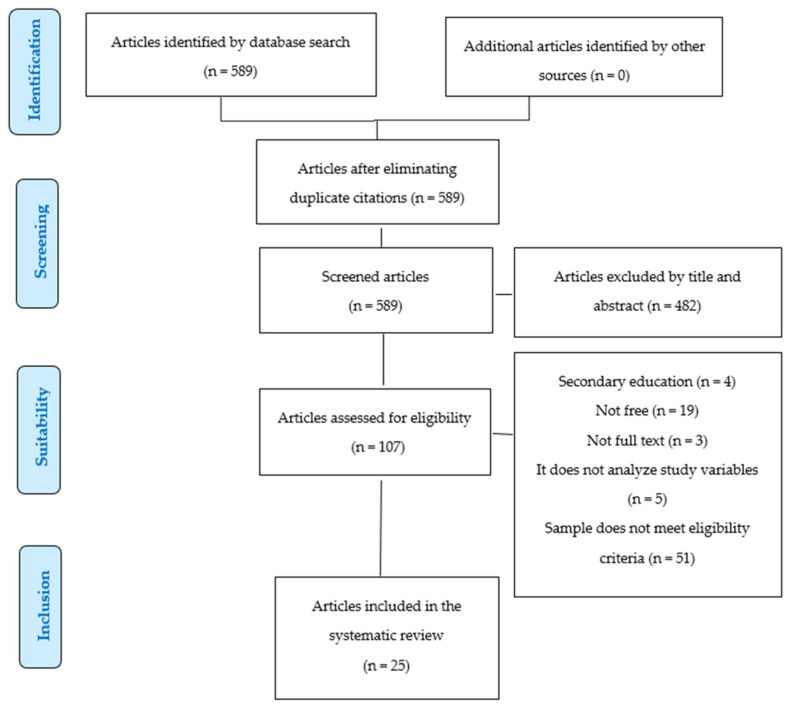
Flowchart for item selection.

A total of 589 articles were found with the search equation and 107 were selected according to the title, which was considered relevant to the research topic. These were analyzed individually; 82 were discarded for not meeting the eligibility criteria to finally select 25 studies, which were analyzed in depth (Figure 1).

### 2.3. Eligibility Criteria

Clinical trials were included with quasi-experimental or pure experimental designs, which were free of charge and were published during the period from January 2010 to December 2020, in Spanish, English or Portuguese language, and conducted in subjects with a diagnosis of T2DM (insulin-requiring or non-insulin-requiring) who were normal weight, overweight or obese, physically inactive and/or sedentary, physically autonomous, and older than 18 years of age.

On the other hand, the exclusion criteria were the following: duplicate publications or substudies of clinical trials, literature reviews, and systematic reviews; studies with private or paid access; studies performed in people without pathologies or in patients with type 1 diabetes or gestational diabetes; studies in pediatric patients; studies with n less than 10; and studies that do not provide information on the glycemic profile of adults with T2DM.

### 2.4. Data Extraction

This procedure was developed with two independent reviewers (B.P.F. & R.V.V.), who analyzed the articles and evaluated the risk of bias according to criteria such as adequate masking and randomization of the participants, complete follow-up of the program, information on balanced losses between groups, and the reporting of concrete results of the pre-specified variables. A data review and extraction form, formulated by consensus, was used. Any discrepancies between reviewers were resolved by mutual agreement.

### 2.5. Synthesis of Results

Double-entry tables were used to synthesize the most relevant information from each article. Regarding the results of the PE programs, a *p*-value < 0.05 was established to measure significant differences.

## 3. Results

From a total of 107 articles analyzed in this systematic review, 25 were selected for meeting the inclusion criteria. The PE methods found were: aerobic exercise (AE), interval exercise (IE), resistance exercise (RE), combined exercise (COM), and other specific methods that differed in protocol and modality from those mentioned, and were therefore classified as “other” (Table 1).

In total, 68% of the selected studies consisted of a short-term program, i.e., of less than 12 weeks’ duration, while the remaining 32% were equal to or longer than that period. Likewise, 28% of the articles established glycemic control as the only study variable, while the remaining 72% simultaneously incorporated the body composition variable (Table 1).

The classification of the PE methods was made on the basis of their modality and the organization of components such as duration, intensity, and volume (Table 2).

### 3.1. Overall Analysis of Results

The present systematic review involved 1128 adult subjects diagnosed with T2DM, ranging in age from 30 to 85 years, with an average age of 57.4 years. In total, 54% of the participants were men and 46% were women, all physically inactive and were either using oral antidiabetic drugs or exogenous insulin. A total of 84% (n = 21) of the studies did not stop the intake of medications. In addition, only two studies considered subjects with normal BMI [10,11], while the rest involved overweight or obese individuals. About 40% (n = 10) of the studies included subjects with uncontrolled T2DM, i.e., with an HbA1c level equal to or higher than 7%, while the remaining 60% (n = 15) only considered patients with T2DM controlled (who are receiving various anti-diabetic treatments).

### 3.2. Synthesis of the Selected Articles

In total, 76% of the articles reviewed presented significant improvements in glycemic control and 78% in body composition; however, there were methods that reported effectiveness more frequently (Table 3). We found that COM achieves significant improvements in all its interventions. The AE achieves greater reductions in HbA1c when it is maintained for more weeks. The combination RE + AE provides longer lasting benefits, while with RE + IE the improvements in glycemic control are attenuated in the medium term and only those in body composition are maintained. Other PE methods offer alternatives for the treatment of T2DM: *sitting less* provides benefits in acute glycemic control, SE and WBVE improves HbA1c, and the recreational practice of soccer is also an effective alternative for the treatment of T2DM and improving body composition.

Other PE methods offer alternatives for the treatment of T2DM: *sitting less* provides benefits in acute glycemic control similar to structured AE; and SE improves HbA1c, as does WBVE, of which more studies are needed to verify its influence on glycemic parameters. On the other hand, REHIT does not provide benefits.

The recreational sports practice of soccer is also an effective alternative for the treatment of T2DM.

### 3.3. Effects of Aerobic Exercise

In total, 56% (n = 10) of the programs with AE were effective in glycemic control, of which 70% (n = 7) were short term, improving acute parameters such as MGC, PPG, and FG, while the remaining 30% (n = 3) were long term and reduced the HbA1c. Regarding body composition, 64% (n = 7) of the interventions reported significant improvements.

Modalities ranged from walking, jogging, cycling, and soccer small-sided.

### 3.4. Effects of Interval Exercise

In total, 67% (n = 6) of the interventions with IE showed significant benefits in glycemic control (*p* < 0.01 to *p* < 0.001), demonstrating effectiveness in the short (one session) and long term (16 weeks), while 80% (n = 4) showed improved body composition, in BMI, BW, SF, TFM, MM, through programs of 11 or more weeks.

Modalities of walking and pedaling on a cycle ergometer were used. In total, 84% (n = 5) of the interval walking interventions significantly improved glycemic control. The effective protocols were developed in 3 min intervals at high velocity and 3 min of active low-effort recovery [10,11,20,24,31], while the only program with 1 min intervals was ineffective [10], despite having equal volume, intensity, and caloric expenditure. These results suggest that reducing the duration of the interval, while keeping the speed and volume dedicated to a fast and slow walking constant, does not produce additional improvements. Likewise, all IE interventions with pedaling on a cycle ergometer benefited body composition; however, glycemic control only improved with intervals of 1 min of high intensity and 1 min of active recovery [29], while there was no change with longer recoveries (3 min) [21], nor with lower-intensity sets [17].

### 3.5. Effects of Resistance Exercise

RE was the least frequent method in this review, comprising only 7% (n = 3) of the total interventions. Of these programs, 33% (n = 1) were effective on glycemic control and 66% (n = 2) on body composition. The transition from medium to high intensity did not yield changes in any study variable [13], while at an intensity of 70 and 80% of 1RM reported improvements [15]. Likewise, in the long term (36 weeks), RE only presented changes in body composition, since to improve glycemic control, it was combined with AE.

### 3.6. Effects of Combined Exercise

The COM method demonstrated significant improvements in both study variables in 100% of its interventions (n = 9), being effective in the short (8 weeks) and long term (36 weeks) and in AE + RE and IE + RE modalities, with no significant differences reported between both combinations [30]. On the other hand, after comparing COM versus AE and RE separately, better results in both variables were reported with the combination [12].

### 3.7. Effects of Other Physical Exercise Methods

In total, 60% (n = 3) of the interventions with other PE methods reported significant improvements in glycemic control and 50% (n = 2) in body composition.

The “breaking sitting with light activities” method [25] reduced and controlled 24 h MGC levels, in 4 days of intervention, with a protocol that consisted of decreasing sedentary behaviors, replacing them with low-intensity activities, such as standing and slow walking, and was compared with an AE intervention, proving to be an equally effective alternative for glycemic control as structured aerobic PE. The SE [33] was compared with the WBVE method using similar protocols, with the difference that the latter was performed on a vibrating platform with a frequency of 12 to 18.5 Hz. Both interventions reduced HbA1c, however, only the WBVE improved body composition. Likewise, there was another study with a similar WBVE [34] that did not report changes in any variable.

Finally, no significant changes were found with the REHIT [27] method.

## 4. Discussion

The main objective of this systematic review was to investigate the literature on the effects of different PE methods on glycemic control and body composition in adults with T2DM, and to define how PE components affect the treatment of this disease. The findings indicate that PE is effective in the glycemic control and body composition of adults with T2DM, by means of methods such as AE, IE, RE, COM, and other less traditional methods, both in the short and long term, improving acute and chronic glycemic parameters (mainly HbA1c), respectively. In addition, it is emphasized that the adequate organization of the components of physical exercise, such as frequency, duration, volume, and intensity, are directly related to the results obtained.

The findings indicate that PE is effective in the glycemic control and body composition of adults with T2DM, coincide with theoretical precedents [6,7,8,35] suggest that PE causes physiological adaptations that improve plasma glucose stabilization and favor the control of chronic hyperglycemia, in addition to body composition, as a result of skeletal muscle remodeling at the cellular level that increases basal metabolism and oxidative capacity [36]. The relevance of improving the body composition of subjects with T2DM is due to the fact that this favors insulin sensitivity [37]. Likewise, PE interventions that report body weight loss have greater glycemic control benefits than those that do not [38]. In this aspect, it is important to note that improvements in body composition occur in studies lasting longer than 8 weeks.

According to the results of the present review, the benefits of PE are achieved by different methods, and they are short and long term. In short-term studies, parameters such as PPG, MGC, and FG were improved, while in long-term studies the main indicator was HbA1c. The latest ADA guidelines [5] determine that there is no indicator superior to another for the diagnostic criteria of T2DM, since each one detects this pathology in different types of patients. However, for purposes of measuring glycemic control and the risk of complications associated with T2DM, the gold standard is the HbA1c, and aims to maintain percentage levels below 7%.

With respect to the PE methods found in this review, AE presented greater controversy, since there were programs with similar characteristics (duration, frequency, intensity, volume, and modality) that differed in their results [11,12,13,14,15,16,17,18,20,21,22,23,24,27,28,29,30,31,32]. Therefore, it is complex to define the factors that could influence; however, it is known that an optimal level of aerobic fitness decreases HbA1c and mortality in patients with T2DM [39]. Likewise, the literature indicates that these benefits are greater when AE is performed for more than 150 min per week [40]; however, in the present review this could not be proven, since the study with AE that reported a greater reduction in HbA1c (↓ 1.84%) [26] varied its weekly volume between 120 and 150 min, while there was another of up to 300 min that reported no changes [11].

Regarding the frequency of AE, general recommendations [1,5] suggest that it should be at least three times per week on nonconsecutive days. In the present review, one study [23] prescribed a weekly frequency of only two days, likewise reporting improvements in HbA1c and body composition.

Among the modalities with AE found in this review, the practice of soccer small-sided is highlighted [23], which was an effective alternative for glycemic control and body composition in men with T2DM. In addition, this sport facilitates psychosocial interactions, which can stimulate the development of physical activity habits consistently over time [41]. Therefore, it is advisable to increase sports interventions to offer alternatives for the treatment of T2DM.

RE was the least frequent method in this review, despite being incorporated in the general recommendations for T2DM [2,5]. Its importance could be explained by the inverse correlation between increased muscle mass and decreased HbA1c [42]. It is estimated that the low use of RE is due to the complexity of its practice, since its performance requires a good execution technique, especially in untrained subjects, who are more prone to joint and muscle injuries [43]. In a study analyzed in this review [13], eight participants reported injuries, precisely in a home-based RE program, in which professional supervision was not constant. In addition, the lowest adherence of an experimental group (71%) of this entire review was reported.

Chulvi-Medrano and Muñoz [44] indicate that RE treatments should be complemented with cardiovascular exercise. In this review, there were nine interventions [12,16,19,28,30] with the COM method, of which eight were AE + RE; all reported effectiveness in both study variables. Likewise, a study [19] compared two similar AE + RE programs and showed that the higher the intensity of RE, the greater the benefits in glycemic control, at least in short-duration programs. On the other hand, another study in this review [28] reported that, in the long term, this combination causes similar effects, regardless of the volume and intensity of the exercises.

Therefore, it is suggested that the COM programs be adapted according to the motivations of the patients, considering that most of them do not tolerate the effort of vigorous PE or perceive it as more demanding [45]; thus, a longer and lower-intensity program is recommended for them, while, on the other hand, there are patients who are better motivated with exercises of greater effort [10,27].

Another method analyzed in this review was IE, frequently used in the clinical area during the last decade. It proposes a more time-efficient alternative [36], which coincides with the fact that 83% of the effective programs in this review were of short duration. Furthermore, 86% of the comparative studies between IE and AE [11,17,20,21,24,29,31] reported better results with intervals, even with similar or lower session volumes than aerobic.

From the methodological point of view, IE facilitates metabolic and cardiovascular adaptations in untrained subjects since it provokes a greater stimulus at the physiological level by making it possible to perform varied periods of vigorous intensity within the same session [46]. In addition, the recovery periods between sets favor the reduction of lactate and cause a greater energy expenditure, as a result of the increase in myoglobin [47].

Studies indicate that the greater the intensity of PE, the greater the benefits of glycemic control [48,49], which is similar to the study by Winding et al. [29], analyzed in this review. The theoretical explanation lies in the rapid depletion of muscle glycogen stores, which allows the activation of cellular sensors regulating mitochondrial biogenesis [48]. Likewise, Little et al. [35] indicated that high-intensity IE could increase GLUT4 protein content by up to 369%, significantly improving glucose uptake capacity and insulin sensitivity. However, in the present review, a study [10] compared two groups of IE of equal intensity and volume, reporting improvements in glycemic control only in the 3:3 min interval group and not in the 1:1 group, demonstrating that the duration of the intervals is more important than the intensity.

Among the limitations of high-intensity IE, the ADA (2019) notes that it presents risks and contraindications in people with T2DM, mainly diue to events of hyperglycemia, hypoglycemia, dehydration, and musculoskeletal injuries being more prone in these interventions. However, a study analyzed in the present review [31] demonstrated the opposite; a high-intensity walking program, in which post-PE glycemia decreased in an attenuated manner, proved to be effective in preventing hypoglycemic episodes in adults with T2DM.

Regarding the other methods selected in this systematic review, one study [25] showed that replacing sedentary behaviors with standing and slow walking is just as effective for glycemic control as structured EA. Although this finding was short-term and similar studies of longer duration were not found, it still offers a more viable and safer alternative to structured programs, and it achieved better post-PE glucose stabilization than AE, reducing the likelihood of hypoglycemic events. Likewise, it is known that there is a correlation between sedentary lifestyle and markers of metabolic alterations [50], therefore, reducing these behaviors is fundamental for the glycemic control of subjects with T2DM, and dividing sitting time with brief episodes of light walking or strength actions improves postprandial glucose and insulin levels [51].

On the other hand, REHIT did not cause significant effects on any variable [27]. Although this is an adaptation of the HIIT method, which did demonstrate effectiveness, it follows that REHIT has a volume well below that which is recommended [52].

Another method found in this review was the whole-body vibration exercise (WBVE), which, according to authors [53,54], stimulates muscle contraction and causes improvements in peripheral blood flow, energy consumption, muscle strength, and glycemic parameters, so it began to be applied in diabetics with microvascular complications and/or advanced age to reduce the risk of adverse events. However, one study [34] reported that there were no significant changes in any variable with WBVE, while another article [33] demonstrated improvements in HbA1c and FG in a group with WBVE and another with SE, both with respect to a pretest, and no differences were found after comparing them with each other. On the other hand, body composition improved only in the vibration group. This result could indicate that the changes in glycemia variables were an exclusive effect of PE, while those in adiposity were due to WBVE; this is similar to another finding in the literature [55], in which there was no improvement in glycemic control with vibrations either, but they were shown to be effective in reducing fat mass in people with T2DM [56]. Therefore, the need for further clinical trials to clarify the effects of WBVE on glycemic control and body composition is suggested.

The knowledge and results generated by addressing the research objective show that PE is effective in glycemic control and body composition in adults with T2DM. It is strongly believed that developing innovative progressions will open new horizons and research opportunities, with the potential to promote physical activity as an effective and safe means for the treatment of TDM2.

Among the limitations of this systematic review is the exclusion of other databases, which could have provided useful scientific articles for the analysis. Likewise, the absence of a meta-analysis limited the assessment of the results, mainly of findings from comparative studies.

## 5. Conclusions

The research analyzed shows that PE is effective in glycemic control and body composition in adults with T2DM, and means of methods such as AE, IE, RE, COM and other less traditional methods, both in the short and long term, improving acute and chronic glycemic parameters (mainly HbA1c). In addition, studies that improve body composition parameters show greater glycemic control benefits.

In relation to the components of PE, the duration of the programs is fundamental since the greatest benefits in glycemic control are obtained in programs of long duration. In low-volume programs, high-intensity PE is crucial, although the benefits are less lasting than in those that comply with the recommendation of 150 min per week and a frequency of 3 days per week.

The knowledge generated by current research has mainly focused on the effects of AE and IE in adults with T2DM, and the number of investigations with combined exercises (COM) is increasing, so it seems that more research with other methods are still needed, and this would be a breakthrough for the treatment of TDM2.

In view of the different alternatives found, it is suggested that PE programs be adapted based on their method and components, according to the characteristics of the patients, such as age, baseline parameters, physical condition, motivational aspects, and availability. Long-term interventions are also recommended to develop PE habits and maintain the benefits over time.

## Figures and Tables

**Table 1:** Classification of selected articles.

PE Method	Articles (n)	Study Duration		Study Variables	
		Long *	Short **	GC and BC	GC
Aerobic	6	2	4	3	3
Interval	1	0	1	0	1
Resistance	1	1	0	1	0
Combined	5	3	2	5	0
Other	2	0	2	2	0
Aerobic vs. Interval	7	1	6	5	2
Aerobic vs. Resistance	1	1	0	1	0
Aerobic vs. Other	2	0	2	1	1
Total	25	8	17	18	7

Note. GC: glycemic control. BC: body composition. * Long duration: ≥12 weeks. ** short duration: <12 weeks.

**Table 2:** Classification of physical exercise methods.

PE Method	PE Description	Interventions (n)
Aerobic	Moderate/vigorous intensity PE, continuous or in long-duration segments; on treadmill, cycle ergometer and/or elliptical trainer.	18
Interval	PE in intervals of high intensity and short/medium duration, alternating with recovery phases; on treadmill and/or cycle ergometer.	9
Resistance	Anaerobic PE of strength, with and without the use of external weight; with dumbbells or multifunctional machines.	3
Combined	Combination of any of the described methods.	9
Other	Reduced Exertion High Intensity Interval Training (REHIT); Isometric PE based on squats (SE); Whole Body Electrical Vibration PE (WBVE); restriction of sedentary behaviors (sitting less).	5
Total		44

Note. PE: physical exercise.

**Table 3:** Summary of the selected studies.

Author	Sample		Prescription	Results		Conclusion
	n (M/W)	Age (SD)		Variables	Significant Effects	
Church et al., 2010 [12]	262 (97/165)	55.8 (8)	36 weeks. 3 days/wk. AE: 12 kcal/kg per wk; 50–80 VO_2_max. RE: 4 upper body Ex, 3 lower body ex and 2 abdominal-back Ex; 2 sets × 10–12 repetitions. AE + RE: AE × 1 day/wk + RE × 2 days/wk. CG: Stretching and relaxation exercises.	HbA1c (%) TFM (kg) MM (kg)	AE: ↓ MM (−0.5 [* vs. RE y COM]). RE: ↓ TFM (−1.4 [* vs. CG]). AE + RE: ↓ HbA1c (−0.34 [* vs. CG]) and ↓ TFM (−1.7 [* vs. CG y AE]). CG: No change.	A combination of continuous aerobic and resistance PE improves glycemic control in adults with T2DM; not so with separate methods of similar caloric expenditure. Body composition only improves with resistance and combined methods.
	AE: 72 (27/45)	53.7 (9)				
	RE: 73 (30/43)	56.9 (8)				
	AE + RE: 76 (27/49)	55.4 (8)				
	CG: 41 (13/28)	58.6 (8)				
Plotnikoff et al., 2010 [13]	48 (16/32)		16 weeks. 3 days/wk. RE: 8 ex with dumbbells at progressive intensity (2–3 sets × 8–12 repetitions at 50–85% 1RM). CG: No PE.	HbA1c (%) TFM (kg) MM (kg)	No significant effects are reported.	16 weeks of supervised home-based resistance PE does not improve glycemic control or body composition in adults with T2DM.
	RE: 27 (8/19)	55 (12)				
	CG: 21 (8/13)	54 (12)				
Belli et al., 2011 [14]	19 (0/19)		12 weeks. 3 days/wk. AE: Continuous outdoor walking × 20 min (wk 1), 30 min (wk 2), 40 min (wk 3) 50 min (wk 4) and 60 min (wk 5–12) at individual VT speed. CG: No PE.	HbA1c (%) FG (mmol/L) BMI (kg/m^2^) BW (kg) TFM (kg)	AE: ↓ HbA1c (−0.9 [* p-p]), ↓ BMI (−1.1 [* p-p and CG]), ↓ BW (−2.5 [** p-p and CG]) and ↓ TFM (−3.4 [** p-p]). CG: No change.	12 weeks of continuous outdoor aerobic walking PE supports glycemic control and body composition in adult women with T2DM.
	AE: 9 (0/9)	53.4 (2)				
	CG: 10 (0/10)	55.9 (2)				
Bacchi et al., 2012 [15]	38 (26/12)		16 weeks. 3 days/wk. AE: Variety of continuous aerobic activities on treadmill and cycle ergometer × 60 min at 60–65% HRR. RE: 9 ex × 3 sets × 10 repetitions at 70–80% 1RM.	HbA1c (%) FG (mmol/L) BMI (kg/m^2^) TFM (kg) MM (kg)	AE: ↓ HbA1c (−0.4 [*** p-p]), ↓ FG (−0.84, [*** p-p]), ↓ BMI (−0.76 [*** p-p]) and ↓ TFM (−1 [*** p-p]). RE: ↓ HbA1c (−0.35 [*** p-p]), ↓ FG (−0.67 [*** p-p]), ↓ BMI (−0.54 [*** p-p]) and ↓ TFM (−1.71 [*** p-p]).	16 weeks of continuous aerobic or resistance PE are equally effective in improving glycemic control and body composition in adults with T2DM.
	AE: 19 (13/6)	57.2 (1)				
	RE: 19 (13/6)	55.6 (1)				
Gibbs et al., 2012 [16]	112 (69/43)		26 weeks. 3 days/wk. AE + RE: Continuous aerobic exercise × 45 min at 60–90% MHR + RE of 7 Ex × 2 sets × 12–15 repetitions at 50% 1RM. CG: No PE.	HbA1c (%) BMI (kg/m^2^) TFM (%)	AE + RE: ↓ HbA1c (−0.2 ± 1.2 [* vs. CG]), ↓ BMI (−0.7 ± 1.6 [* vs. CG]) and ↓ TFM (−1.4 ± 1.9 [*** vs. CG]). CG: No change.	26 weeks of continuous aerobic PE combined with resistance improves glycemic control and body composition in adults with T2DM.
	AE + RE: 49 (32/17)	58 (5)				
	CG: 63 (37/26)	56 (6)				
Li et al., 2012 [17]	55 (30/25)		12 weeks. 4 days/wk. AE: Aerobic exercise at 50% VO_2_peak, on treadmill: 1 set × 15 min (wk 1), 2 × 15 min (wk 2), 2 × 20 min (wk 3–4) and 2 × 120 kcal (wk 5–12). IE: Exercise similar to AE (wk 1–2); 2 sets × 15 min at 65% VO_2_peak (wk 3–4) and 2 × 120 kcal at 75% VO_2_peak (wk 5–12).	HbA1c (%) FG (mmol/L) BMI (kg/m^2^) TFM (%)	AE: ↓ BMI (−0.6 [* p-p]) and ↓ TFM (−1% [* p-p]). IE: ↓ BMI (−0.5 [* p-p]) and ↓ TFM (−1% [* p-p]).	12 weeks of aerobic or interval PE does not favor glycemic control in adults with T2DM. On the other hand, both improve body composition in equal magnitude.
	AE: 27 (15/12)	52.0 (1)				
	IE: 28 (15/13)	50.3 (1)				
Mikus et al., 2012 [18]	AE: 13 (8/5)	53 (2)	1 week. 7 days/wk. AE (pre): Habitual sedentariness × 3 days. AE (pos): 60 min of aerobic exercise in periods of 20 min of treadmill walking alternated with 20’ of stationary cycling, at 60–75% HRR.	HbA1c (%) MGC (mmol/L) PPG (mmol/L) FG (mmol/L)	AE (pos): ↓ PPG (* p-p).	7 consecutive days of aerobic PE improves postprandial glycemic control in adults with T2DM.
Egger et al., 2013 [19]	32 (13/19)	64 (7)	8 weeks. 2 days/wk. RE_1_ +AE: 6 Ex × 2 sets × 20–30 repetitions at 40% 1RM, with 3–5 min of recovery between sets + AE (60 min of continuous pedaling on a cycle ergometer at 70% HRR). RE _2_+ AE: 6 Ex × 2 sets × 20–30 repetitions at 70% 1RM, with 3–5 min recovery between sets + similar AE.	HbA1c (%) FG (mmol/L) BMI (kg/m^2^) BW (kg) MM (kg) WC (cm)	RE_1_ + AE: ↓ FG (−0.68 [** p-p]), ↓ BW (−0.3 [** p-p]), ↓ BMI (−0.1 [* p-p]), ↑ MM (0.2 [** p-p]) and ↓ WC (−2 [*** p-p]). RE_2_ + AE: ↓ FG (−0.76 [** p-p; * vs. RE_1_]), ↓ BW (−1.4 [** p-p]), ↓ BMI (−0.4 [* p-p]), ↑ MM (0.3 [** p-p]), ↓ WC (−2.1 [*** p-p]).	8 weeks of resistance PE combined with continuous aerobics promotes FG in adults with T2DM. The effect is superior with higher intensity resistance. In terms of body composition, both methods are effective in equal magnitude.
	RE_1_ +AE: 16 (5/11)	64 (7)				
	RE_2_ +AE: 16 (8/8)	65 (8)				
Karstoft et al., 2013 [20]	32 (20/12)		16 weeks. 5 days/wk. AE: 60 min free-living interval-walking training 55% VO_2_peak. IE: 60 min of free-living walking in intervals of 3’ > 70% VO_2_peak and 3’ < 70%. CG: No PE.	HbA1c (%) MGC (mmol/L) FG (mmol/L) BMI (kg/m^2^) BW (kg) TFM (kg MM (kg)	AE: No change. IE: ↓ MGC (−0.7 [* p-p; ** vs. CG]), ↓ BW (−4.2 [*** p-p; ** vs. CG; * vs. AE]), ↓ BMI (−1.4 (* vs. CG y AE; *** p-p]) and ↓ TFM (−3.1 [** vs. CG and AE; *** p-p]). CG: ↑ MGC (1.2 [* p-p]).	High-velocity interval walking is effective in improving glycemic control and body composition in adults with T2DM. On the other hand, aerobic walking does not cause changes and inactivity worsens glycemic parameters.
	AE: 12 (8/4)	60 (2)				
	IE: 12 (7/5)	57 (2)				
	CG: 8 (5/3)	57 (3)				
Terada et al., 2013 [21]	15 (8/7)		12 weeks. 5 days/wk. IE: Exercise on a cycle ergometer and treadmill in intervals of 1 min at 100% VO_2_R × 3 min recovery at 20%, × 30 min (wk 1–4), 45’ (wk 5–8) and 60 min (wk 9–12). AE: Continuous exercise × 30 min (wk 1–4), 45 min (wk 5–8) and 60 min (wk 9–12) at 40% VO_2_R.	HbA1c (%) FG (mmol/L) BMI (kg/m^2^) BW (kg) TFM (%)	IE: ↓ TFM (−1.9% [** p-p]). AE: ↓ TFM (−1.5% [* p-p]).	Neither high-velocity interval PE nor continuous aerobic PE is effective for glycemic control in adults with T2DM. However, both show improvements in adiposity.
	IE: 8 (4/4)	62 (3)				
	AE: 7 (4/3)	63 (5)				
Van Dijk et al., 2013 [22]	60 (60/0)	60 (6)	1 session of 45–60 min AE_IR_: Continuous pedaling on a cycle ergometer for 45–60 min at 35–50% Wmax. AE_NRI_: Method similar to AE_IR_.	MGC (mmol/L)	AE_IR_: ↓ MGC (−0.9 [*** p-p]). AE_NRI_: ↓ MGC (−0.9 [*** p-p]).	One session of continuous aerobic PE is effective for 24 h glycemic control of adult men with insulin-treated and non-insulin-treated T2DM. The effects are greater in uncontrolled subjects (>7% HbA1c).
	AE_IR_: 23 (23/0)	60 (5)				
	AE_NRI_: 37 (37/0)	59 (6)				
	Crossover trial					
Andersen et al., 2014 [23]	21 (21/0)	49.8 (1)	24 weeks. 2 days/wk. AE: 60’ of soccer small-sided (5 Ex × 10 min continuous at 83 ± 2% MHR, with 2 min of passive rest. CG: No PE.	HbA1c (mmol/L) TFM (kg) MM (kg)	AE: ↓ HbA1c (−0.4 (** p-p) and ↓ TFM (−1.7 (*** p-p; ** vs. CG). CG: ↓ MM (−0.5 (* p-p).	Aerobic PE via soccer small-sided is effective in improving glycemic control and adiposity in adult men with T2DM.
	AE: 12 (12/0)					
	CG: 9 (9/0)					
Karstoft et al., 2014 [24]	10 (7/3)	60 (2)	1 session of 60 min AE: Continuous treadmill walking at 73% VO_2_peak. IE: Treadmill walking with intervals of 3 min at 54% and 3 min at 89% VO_2_peak. CG: No PE.	FG (mmol/L) PPG (mmol/L)	AE: No change. IE: ↓ PPG (−1.2 [** vs. CG]). CG: No change.	A session of high-velocity interval walking improves postprandial glycemic control in adults with T2DM. Continuous walking of equal volume and energy expenditure does not.
	Crossover trial					
Jakobsen et al., 2016 [10]	11 (6/5)	61.6 (8)	1 session of 60 min IE_1_: Treadmill walking in intervals of 3 min at 54% VO_2_peak and 3 min at 89% VO_2_peak. IE_2_: Treadmill walking in intervals of 1 min at 54% VO_2_peak and 1 min at 89% VO_2_peak. CG: No PE.	MGC (mmol/L) PPG (mmol/L)	IE_1_: ↓ MGC (* vs. CG), ↓ PPG (−0.8 [* vs. CG]). IE_2_: No change. CG: No change.	high-velocity interval walking is effective in postprandial glycemic control in adults with T2DM only at 3 min intervals. Interval duration is more important than intensity and session volume.
	Crossover trial					
Duvivier et al., 2017 [25]	19 (13/6)	63 (9)	4 consecutive days. “Breaking sitting with light activities”, Replace 4.7 h/day of sitting with 2.5 h/day of standing and 2.2 h/day of light walking. AE: Replace 1h/day of sitting with 3 sets of 20 min of pedaling on a cycle ergometer at 50–60% Wmax. CG: Sedentary regimen of 14 h/day sitting; walking <1 h/day and standing <1 h/day.	MGC (mmol/L)	Breaking sitting with light activities: ↓ MGC (−0.34 [** vs. CG]). AE: ↓ MGC (−0.4 [** vs. CG]). CG: No change.	Replacing sitting time with standing and activities at low intensity is an equally effective alternative to structured aerobic PE for improving 24 h glucose levels in adults with T2DM.
	Crossover trial					
Karstoft et al., 2017 [11]	14 (11/3)	65 (2)	2 weeks. 5 days/wk. AE: 60 min of continuous treadmill at 73% walking peak VO_2_. IE: 60 min of treadmill walking in intervals of 3 min at 54% walking peak VO_2_. and 3 min at 89%. CG: No PE.	HbA1c (%) MGC (mmol/L) FG (mmol/L) BW (kg) TFM (%) MM (kg)	AE: No change. IE: ↓ FG (−0.5 [* vs. CG]), ↓ MGC (−0.7 [* vs. CG; ** p-p]). CG: No change.	2 weeks of high-velocity interval walking improves glycemic control in adults with T2DM. Continuous aerobic walking does not. Both methods do not cause changes in body composition.
	Crossover trial					
Najafipour et al., 2017 [26]	30 (not specified)	57 (8)	16 weeks. 3 days/wk. AE: Continuous aerobic PE on treadmill, exercise or elliptical bike × 40–50 min at 50–80% MHR. CG: No PE.	HbA1c (%) BMI (kg/m^2^)	AE: ↓ HbA1c (−1.84 [*** p-p]) and ↓ BMI (−1.84 [*** p-p]). CG: ↑ BMI (1.83 [* p-p]).	Continuous aerobic PE is effective for glycemic control and body composition in adults with T2DM in the medium (16 weeks) and long term (8 years).
	AE: 15	57 (8)				
	CG: 15	57 (8)				
Ruffino et al., 2017 [27]	16 (16/0)	55 (5)	8 weeks. REHIT: 3 days/wk, 10 min cycling at 25W with 2 sprints of 10 s (sessions 1–4), 15 s (sessions 5–12) and 20 s (sessions 13–24). AE: 5 days/wk, 30 min continuous walking at 40% HRR (wk 1–2), 50% (wk 3–4) and 55% (wk 5–8).	MGC (mmol/L) FG (mmol/L) BW (kg) TFM (%)	No significant effects are reported.	Neither the REHIT method nor continuous aerobic walking is effective in improving short-term glycemic control and body composition in adult men with T2DM.
	Crossover trial					
Yang et al., 2017 [28]	62 (32/30)	52 (1)	24 weeks. RE (2 days/wk); AE (5 days/wk) RE_1_ + AE: 12 weeks of RE (10 ex × 2 sets × 15 repetitions at 50% 1RM) + 12 weeks of AE (30’ of walking/biking at 60–80% HRR). RE_2_ + AE: 24 weeks of RE (10 ex × 3 sets × 7 repetitions at 75% 1RM) + AE (similar). RE_3_ + AE: 24 weeks of RE (similar to RE_1_) + AE (similar).	HbA1c (%) FG (mmol/L) BMI (kg/m^2^) BW (kg) TFM (%) MM (kg)	RE_1_ + AE: ↓ HbA1c (−1.1 [*** p-p]), ↓ BMI (2.5 [* p-p]) and ↓ TFM (−2.8 [*** p-p]). RE_2_ + AE: ↓ HbA1c (−1 [*** p-p]) and ↓ TFM (−2.5 [*** p-p]). RE_3_ + AE: ↓ HbA1c (−0.5 [*** p-p]), ↓ BMI (−1.8 [* p-p]) and ↓ TFM (−4.8 [*** p-p]).	Resistance PE combined with continuous aerobic exercise improves glycemic control and body composition in adults with T2DM in the long term. The effect is independent of volume and intensity.
	RE_1_ + AE: 20 (14/6)	52 (1)				
	RE_2_ + AE: 20 (8/12)	49 (1)				
	RE_3_ + AE: 22 (10/12)	54 (1)				
Winding et al., 2018 [29]	32 (19/13)		11 weeks. 3 days/wk. AE: Continuous pedaling × 40 min at 50% HRR. IE: 10 intervals of 1 min of pedaling at 95% HRR × 1 min al 20%. CG: No PE.	HbA1c (%) MGC (mmol/L) PPG (mmol/L) FG (mmol/L) BMI (kg/m^2^) BW (kg) TFM (kg) MM (kg)	AE: ↓ MGC (−0.6 [* p-p]) and ↓ BW (−1 [* vs. CG]). IE: ↓ HbA1c (−0.1 [* p-p]), ↓ FG (−0.7 [* p-p]), ↓ BW (−1.2 [* vs. CG; * p-p]) and ↓ BMI (−0.3 [* p-p]). CG: No change.	11 weeks of continuous aerobic or high-intensity interval PE improves glycemic control and body composition in adults with T2DM.
	AE: 12 (7/5)	58 (8)				
	IE: 13 (7/6)	54 (6)				
	CG: 7 (5/2)	57 (7)				
Womgoor et al., 2018 [30]	23 (23/0)		12 weeks. 3 days/wk. AE + RE: Continuous pedaling × 10 min (wk 1–3); 17,5 min (wk 4–7); and 26 min (wk 8–12) at 55% HRR + RE of 5 ex × 2 sets × 15 repetitions at 67% 1RM (wk 1–3); 3 sets × 10 repetitions at 75% (wk 4–7); and 2 sets × 12 repetitions at 75%. IE + RE: Continuous pedaling × 10 min at 50%HRR (wk 1–3); 3 intervals of 3.5 min at 75%HRR × 3.5 min at 45% (wk 4–7); and 12 intervals of 1 min at 95%HRR × 1 min at 40% (wk 8–12) + RE similar to the group with AE.	HbA1c (%) BMI (kg/m^2^) WC (cm) SF (mm)	AE + RE: ↓ HbA1c (−0.8 [** p-p]), ↓ WC (−2 [** p-p]) and ↓ SF (−29 [*** p-p]). IE + RE: ↓ HbA1c (−0.5 [** p-p]), ↓ WC (−0.5 [** p-p]) and ↓ SF (−25 [*** p-p]).	Resistance PE, in combination with continuous aerobic or high-velocity interval exercise, improves glycemic control and adiposity in men with T2DM, in the short term. These benefits are maintained in the medium term only in adiposity.
	AE + RE: 11	52 (7)				
	IE + RE: 12	52 (7)				
Mendes et al., 2019 [31]	15 (7/8)	60.2 (3)	1 session of 40 min AE: 30 min of continuous treadmill walking at 50% HRR. IE: 30 min of treadmill walking in intervals of 3’ at 70% HRR × 3’ at 30%. CG: 40 min in sedentary position.	MGC (mmol/L)	AE: ↓ MGC (−3.7 [*** vs. CG]). IE: ↓ MGC (−4.1 [*** vs. CG; ** vs. AE]). CG: No change.	Both continuous walking and high-velocity interval walking are effective for acute glycemic control in adults with T2DM. Upon comparison, interval has better results.
	Crossover trial					
Rees et al., 2019 [32]	63 (29/34)	64 (8)	1 session of 50 min AE: Continuous walking at 5 km/h pre-dinner. CG: No pre-dinner PE.	MGC (mmol/L) PPG (mmol/L)	AE: ↓ MGC (−1.56 [*** vs. CG]). CG: No change.	A continuous aerobic walking session before dinner is not effective for either postprandial or 24 h glycemic control in adults with T2DM.
	Crossover trial					
Domínguez-Muñoz et al., 2020 [33]	90 (not specified)	40–85	8 weeks. 3 days/wk. SE: 5–9 1 min isometric squats, with knee flexion at 45° × 30 s recovery. WBVE: Same protocol, but whole-body vibration frequency of 12.5–18.5 Hz was added.	HbA1c (%) FG (mmol/L) BMI (kg/m^2^) TFM (%) MM (%)	SE: ↓ HbA1c (−0.19 [** p-p]), ↓ FG (−0.83 [*** p-p]) and ↑ BMI (1.28 [* p-p]). WBVE: ↓ HbA1c (−0.24 [*** p-p]), ↓ FG (−0.58 [* p-p]), ↓ BMI (−1.94 [** p-p]) and ↓ TFM (−0.67 [* vs. CG; ** p-p]).	8 weeks of physical exercise based on isometric squats improves glycemic control in adults with T2DM. In terms of body composition, there are only improvements when incorporating whole-body vibrations.
	SE: 45					
	WBVE: 45					
Manimmanakorn et al., 2017 [34]	36 (13/23)		12 weeks. 3 days/wk. WBVE: 2 sets × 6 isometric squats of 1 min with vibration frequency of 40 Hz, ×20 s recovery. CG: No PE.	HbA1c (%) FG (mmol/L) BMI (kg/m^2^) BW (kg)	No significant effects are reported.	12 weeks of whole-body vibration exercise does not result in changes in glycemic control or body composition in adults with T2DM.
	WBVE: 17 (7/10)	60.9 (1)				
	CG: 19 (6/13)	63.9 (4)				

Note. ↑: increase; ↓ decrease; p-p: pre-post intervention; ns: not significant; *: *p* < 0.05; **: *p* < 0.01; ***: *p* < 0.001. M: man; W: women; SD: standard deviation; CG: control group; AE: aerobic exercise; IE: interval exercise; RE: resistance exercise; REHIT: reduced exertion high intensity interval training; SE: squat exercise; WBVE: whole body vibration exercise; IR: insulin requiring; NRI: non-requiring insulin; PE: physical exercise; Ex: exercises; Reps: repetitions; Wk: week; FG: fasting glucose; HbA1c: glycosylated hemoglobin; MGC: mean glucose concentration; PPG: post-prandial glycemia; WC: waist circumference; BMI: body mass index; BW: body weight; MM: muscle mass; SF: subcutaneous folds; TFM: total fat mass; HRR: heart rate reserve; MHR: maximum heart rate; 1RM: maximum repetition; VO_2_max: maximum volume of oxygen; VO_2_peak: oxygen pick volume; VO_2_R: reserve oxygen volume; VT: ventilatory threshold; Wmax: maximum power. The HbA1c figures expressed in mmol/l were transformed to %. The figures of MGC, FG, and PPG expressed in mg/dl were transformed to mmol/L.

## Data Availability

The data presented in this study are available on request from the corresponding author.
